# Triazolopyrimidines Target Aerobic Respiration in Mycobacterium tuberculosis

**DOI:** 10.1128/aac.02041-21

**Published:** 2022-03-09

**Authors:** Catherine Shelton, Matthew McNeil, Renee Allen, Lindsay Flint, Dara Russell, Bryan Berube, Aaron Korkegian, Yulia Ovechkina, Tanya Parish

**Affiliations:** a TB Discovery Research, Infectious Disease Research Institute, Seattle, Washington, USA; b Center for Global Infectious Disease Research, Seattle Children's Research Institute, Seattle, Washington, USA

**Keywords:** aerobic respiration, antibiotic resistance, antibiotic tolerance, tuberculosis

## Abstract

We previously identified a series of triazolopyrimidines with antitubercular activity. We determined that Mycobacterium tuberculosis strains with mutations in QcrB, a subunit of the cytochrome *bcc-aa3* supercomplex, were resistant. A cytochrome *bd* oxidase deletion strain was more sensitive to this series. We isolated resistant mutants with mutations in Rv1339. Compounds led to the depletion of intracellular ATP levels and were active against intracellular bacteria, but they did not inhibit human mitochondrial respiration. These data are consistent with triazolopyrimidines acting via inhibition of QcrB.

## TEXT

**FIG 1:**
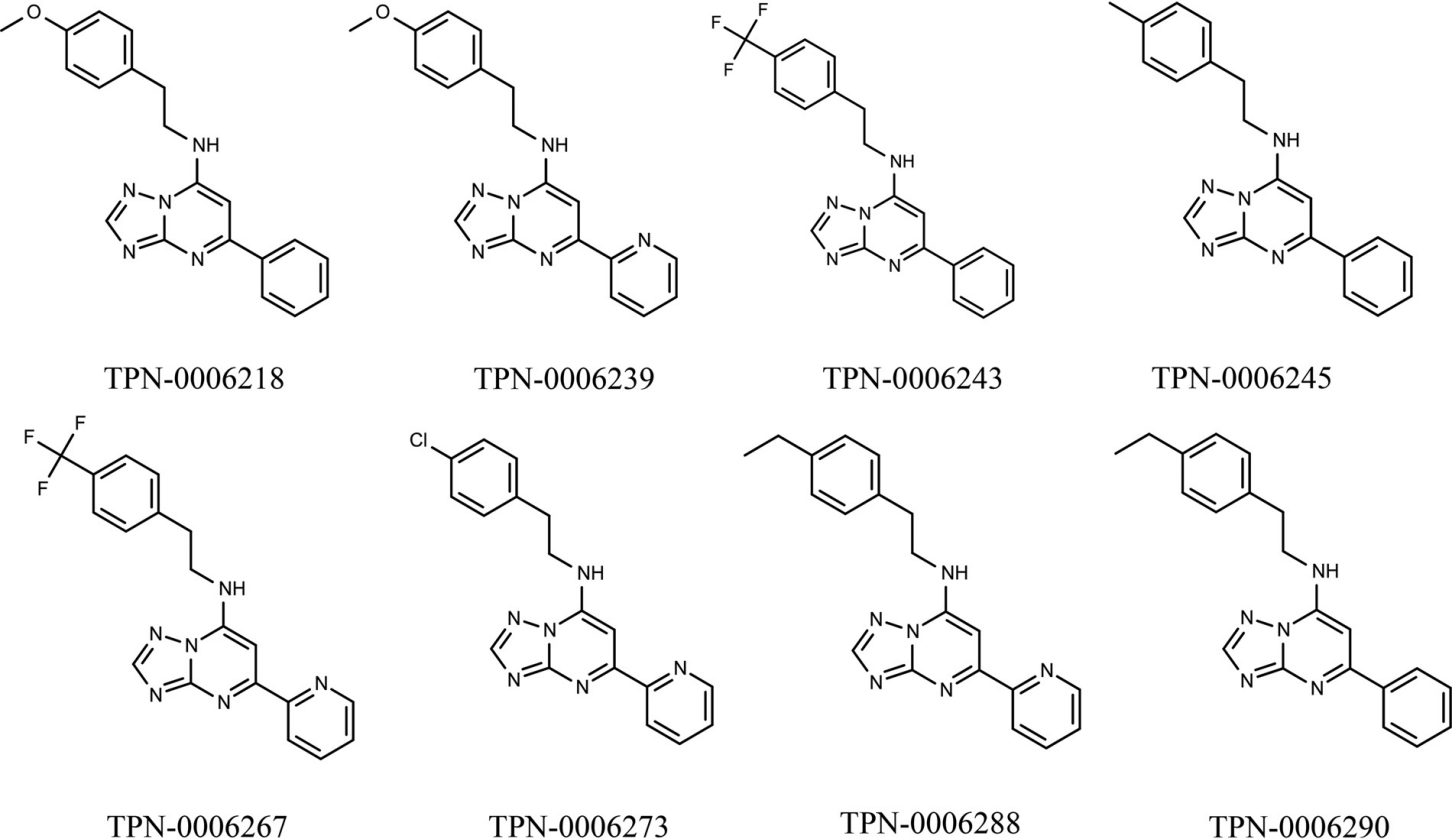
Structures of molecules.

We previously identified a series of triazolopyrimidines with antitubercular activity (1); compounds were bacteriostatic for replicating Mycobacterium tuberculosis but bactericidal for nonreplicating bacteria. We explored the structure-activity relationship and determined druglike properties. We wanted to determine the target and/or mechanism of action of the piperacillin-tazobactam (TZP) series. Since previous work in our group and others had identified several common targets, we tested a set of analogs for activity against strains carrying mutations in promiscuous targets (Fig. 1) (2).

We selected DprE1, MmpL3, and QcrB as the most common targets (3–6). We determined activity against strains carrying either DprE1_C387S_, MmpL3_F255L_, or QcrB_A396T_ mutations in the parental strain M. tuberculosis H37Rv-LP (ATCC 25618) (Table 1) (7). MICs were determined as described after 5 days of growth in Middlebrook 7H9 medium plus 10% (vol/vol) oleic acid-albumin-dextrose-catalase (OADC) supplement and 0.05% (wt/vol) Tween 80 and were determined using a 10-point, 2-fold serial dilution series of each compound (8). We saw a small shift in MICs in the QcrB_A396T_ mutant strain, namely, an ∼2- to 4-fold increase in resistance. No significant changes were seen in the DprE1_C387S_ or MmpL3_F255L_ mutant strains.

**TABLE 1:** Activity against strains of M. tuberculosis

Molecule	MIC^*a*^ (μM) of:			
	Parental (*n*)	DprE1 C387S	MmpL3 F255L	QcrB A396T
TPN-0006218	2.6 ± 1.3 (9)	0.94 ± 0.67	2 ± 0.99	6.8 ± 2.8
TPN-0006239	1.1 ± 0.5 (10)	0.42 ± 0.07	0.86 ± 0.21	3.4 ± 0.28
TPN-0006243	3.7 ± 2.9 (14)	0.95 ± 0.29	2.7 ± 2.4	5.7 ± 2.1
TPN-0006245	2 ± 1.1 (11)	0.92 ± 0.3	2 ± 0.07	5.6 ± 2.5
TPN-0006267	1.4 ± 1.4 (8)	0.66 ± 0.01	1.8 ± 0.57	5.9 ± 3.5

a MICs were determined after 5 days in two independent experiments (except for parental where *n* is the number of independent biological replicates). The genotype of the strain is noted. The parental strain is M. tuberculosis H37Rv-LP (ATCC 25618).

In order to confirm that the QcrB mutation did lead to resistance and is the likely target, we tested compounds against two additional strains carrying QcrB mutations (T313I and M342T) (5, 9). We determined MICs after 5 days of growth in Middlebrook 7H9 medium plus 10% (vol/vol) OADC supplement and 0.05% (wt/vol) Tween 80. QcrB_T313I_ is the most common mutation which confers resistance to inhibitors (Table 2). We confirmed high-level resistance in both strains.

**TABLE 2:** Activity against strains of M. tuberculosis

Molecule	MIC^*a*^ (μM) of:			
	H37Rv ATCC 26518		H37Rv ATCC 27294	
	QcrB_T313I_	QcrB_M342T_	Parental	*cyd*KO
	>20	>20	5.9 ± 0.6	0.38 ± 0.04
TPN-0006239	>20	11	9.5 ± 3.5	0.13 ± 0.007
TPN-0006243	>20	>20	2.2 ± 0.9	<0.39
TPN-0006245	>20	>20	nd	nd
TPN-0006267	>20	10	nd	nd

a MICs were determined after 5 days. The genotype of the strain and parental strain is noted. nd, not determined.

QcrB is a component of the *cytochrome bc_1_* complex in the electron transport chain; M. tuberculosis strains in which the alternative cytochrome oxidase (cytochrome *bd*) is deleted are hypersusceptible to QcrB inhibitors (10, 11). We also tested three compounds against M. tuberculosis H37Rv ATCC 272942 and the isogenic CydC deletion strain (11). As expected, M. tuberculosis H37Rv ATCC 27294 was more resistant to the compounds than H37Rv ATCC 25618, as has been noted with other QcrB inhibitors, although the mechanism behind this is unknown (10–12). Deletion of cytochrome *bd* activity resulted in higher sensitivity to the three compounds (Table 2). Taken together, these data strongly support the hypothesis that the target of the series is QcrB.

**FIG 2:**
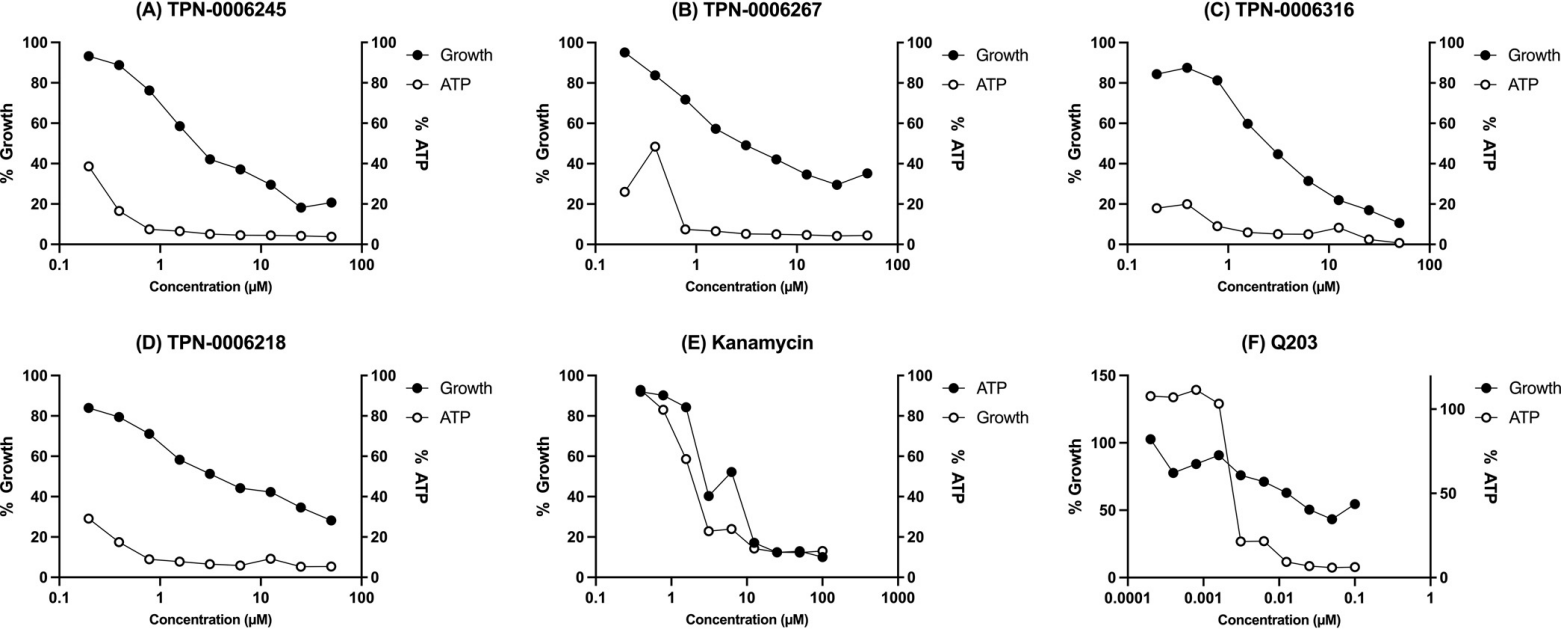
TZP molecules lead to the depletion of intracellular ATP levels. ATP levels were measured in M. tuberculosis using the BacTiter Glo assay kit; growth was measured by OD_590_. Data were normalized to the untreated control (dimethyl sulfoxide [DMSO] only).

We have previously demonstrated that QcrB inhibitors lead to the depletion of intracellular ATP that is independent of the inhibition of growth and is consistent with disruption of the electron transport chain. We determined the effect of compounds on ATP levels (Fig. 2). M. tuberculosis was exposed to compounds for 24 h; ATP levels were measured using the BacTiter-Glo assay kit (Promega). Growth was measured by the optical density at 590 nm (OD_590_). Q203 caused depletion of ATP levels at concentrations lower than the MIC (Fig. 2F). Similarly, ATP levels were reduced in a dose-dependent fashion on exposure to TZP molecules at concentrations which did not inhibit growth (Fig. 2A to D). Depletion of ATP was not seen with the protein synthesis inhibitor kanamycin (Fig. 2E). These data further support the disruption of the electron transport chain as the mechanism of action of the TZP series.

We wanted to determine if there were additional targets or mechanism(s) of resistance, so we isolated and characterized mutants resistant to the series. We selected compounds from our original set with the lowest liquid MIC and determined the MIC against M. tuberculosis H37Rv ATCC 25618 on solid medium (Table 3). We selected two compounds and plated ∼10^8^ bacteria onto 5× and 10× solid MIC as described (4). We isolated colonies and confirmed resistant mutants by measuring the MIC on solid medium; we obtained nine resistant isolates for TPN-0006239 and five resistant isolates for TPN-0006267 (Table 3).

**TABLE 3:** Characterization of resistant isolates of *M. tuberculosis*

Strain	Compound	MIC^*a*^ (μM)	Genotype of:^*b*^	
			Rv1339	QcrB
H37Rv-LP	TPN-0006239	1.6	wt	wt
LP-0497553-RM1	TPN-0006239	25	P121L	wt
LP-0497553-RM2	TPN-0006239	25	P121L	wt
LP-0497553-RM4	TPN-0006239	50	P121L	wt
LP-0497553-RM5	TPN-0006239	50	P121L	wt
LP-0497553-RM10	TPN-0006239	50	S120P	wt
LP-0497553-RM11	TPN-0006239	50	P121L	wt
LP-0497553-RM14	TPN-0006239	50	wt	wt
LP-0497553-RM15	TPN-0006239	50	P121L	wt
LP-0497553-RM23	TPN-0006239	50	wt	wt
H37Rv-LP	TPN-0006267	1.6	wt	wt
LP-0499227-RM1	TPN-0006267	>100	P121L	wt
LP-0499227-RM2	TPN-0006267	>100	P121L	wt
LP-0499227-RM3	TPN-0006267	>100	P121L	wt
LP-0499227-RM4	TPN-0006267	25	P121L	wt
LP-0499227-RM7	TPN-0006267	>100	P121L	wt

a MICs were determined in 24-well agar plates after 3 weeks of incubation. Two genes (*qcrB* and *rv1339*) were sequenced in all strains.

b wt, wild type.

We sequenced the entire QcrB gene in all 14 isolates, but none of them had mutations (Table 3). We had previously seen mutations in Rv1339 leading to resistance to other QcrB inhibitors (5, 9), so we sequenced Rv1339. We found the same mutation in 11 strains (P121L); 1 strain had the mutation S120P (Table 3). Two strains had no mutations in Rv1339. We have previously linked Rv1339 mutations to resistance to other QcrB inhibitor series, namely, the imidazopyridines and the phenoxyalkylimidazoles (5, 9). Recent work in the related organism Mycobacterium smegmatis suggests that Rv1339 is an atypical class II cAMP phosphodiesterase that has been linked to antibiotic tolerance (13). In addition, a P94L mutation in Rv1399 led to increased persistence in animal models and increased resistance to external stress in Mycobacterium canetti, which was proposed to be due to changes in cell wall permeability (14). It is possible that the mutations we obtained lead to decreased compound permeation leading to resistance. However, it is unusual that resistance is seen largely with QcrB inhibitors, not as a general phenomenon; an alternative explanation for resistance could be changes in the intracellular ATP pool due to decreased turnover of cAMP.

We had demonstrated previously that this series had bacteriostatic activity against replicating M. tuberculosis but bactericidal activity against nonreplicating bacteria (1). We have noted this biological activity profile for other QcrB inhibitors, and thus, it is consistent with it being an inhibitor of aerobic respiration (5, 9, 12). Since other QcrB inhibitors are active against intracellular bacteria, we tested the TZP series for activity against M. tuberculosis in human THP-1 macrophages. Macrophages were infected with M. tuberculosis expressing luciferase (15) at a multiplicity of infection of ∼1 for 24 h, washed to remove extracellular bacteria, and then exposed to the compound for 72 h. Bacterial growth was measured by fluorescence. We tested five representative molecules, and all had potent activity with an 50% inhibitory concentration (IC_50_) of <1 μM (Table 4).

**TABLE 4:** Activity against intracellular M. tuberculosis

Molecule	Intracellular IC_50_^*a*^
TPN-0006218	0.23 ± 0.08
TPN-0006267	0.21 ± 0.11
TPN-0006273	0.19 ± 0.13
TPN-0006288	0.076 ± 0.032
TPN-0006290	0.18 ± 0.09

a IC_50_s were measured after 72 h in THP-1 cells infected at a multiplicity of infection of 1 (*n* = 2).

Since we identified the target of the TZP series as aerobic respiration, we determined whether the series might also inhibit mitochondrial respiration. We determined cytotoxicity against HepG2 cells cultured in Dulbecco’s modified Eagle’s medium (DMEM) with galactose as the carbon source to force the cells into using mitochondrial respiration (16). HepG2 cells were exposed to the compound for 72 h, and viability was measured using CellTiterGlo (Promega) (1). Of eight compounds, six showed some cytotoxicity (Table 5), although they still had a good selectivity index (activity was more potent against M. tuberculosis). We compared the IC_50_s under this condition to those generated when HepG2 cells were cultured in glucose when mitochondrial respiration is not active (1). There was less than a 2-fold difference in the cytotoxicity, confirming that molecules are not inhibiting eukaryotic respiration.

**TABLE 5:** Cytotoxicity against human HepG2 cells^*a*^

Molecule	IC_50_^*b*^ (μM)	
	Glucose	Galactose
TPN-0006218	>100	65
TPN-0006239	>100	73
TPN-0006243	>100	>100
TPN-0006245	58	39
TPN-0006267	>100	>100
TPN-0006273	100	76
TPN-0006288	44	23
TPN-0006290	49	33

a HepG2 cells were cultured in medium containing either galactose or glucose as the carbon source.

b IC_50_, the concentration required to reduce cell number by 50%, was determined after 3 days of exposure to compounds.

In conclusion, we have determined that the most likely target of the triazolopyrimidine series in M. tuberculosis is QcrB, a component of the electron transport chain. We demonstrated that mutations in either the target QcrB or the putative phosphodiesterase Rv1339 lead to resistance. This information adds another series of interest to the list of known or proposed QcrB inhibitors, which include the imidazopyridine amides (9), Q203 (17), lansoprazole (18), phenoxyalkylimidazoles (5), morpholino thiophenes (6), quinolinyl acetamides (19), pyrazolopyridines (20), and arylvinylpiperazine amides (21). Since QcrB is a clinically validated target (22), this series is an attractive one to develop further.
